# Oxysterols in stored powders as potential health hazards

**DOI:** 10.1038/s41598-021-00636-5

**Published:** 2021-10-27

**Authors:** Sylwia Chudy, Joanna Teichert

**Affiliations:** Department of Dairy and Process Engineering, Faculty of Food Science and Nutrition, Poznań University of Life Sciences, ul. Wojska Polskiego 31, 60-624 Poznan, Poland

**Keywords:** Biogeochemistry, Health care, Risk factors, Engineering

## Abstract

Cholesterol oxidation products (COPs) have greater biological activity than cholesterol itself. Oxysterols reduce the nutritional value of foods and exhibit a wide range of biological activity, including pro-oxidant, carcinogenic, and cytotoxic properties. The most commonly detected oxysterols in foods are 7α-HC, 7β-HC, a product of their dehydrogenation 7-KC and α-CE, β-CE. The main dietary sources of oxysterols are eggs and egg-derived products, thermally processed milk and milk-based products, fried meat. This study aimed to measure the amount of cholesterol oxidation products in milk powder, egg powder and milk–egg powder during 24 months of storage. The changes in the selected oxysterols (determined by gas chromatography) were recorded. In milk powder, after the production process, the amount of cholesterol was 0.2 g 100 g^−1^ fat and in egg powder it was 3.4 g 100 g^−1^. After 6 months of storage, the dominant oxysterol in milk and egg powder was 7α-HC and in milk–egg powder it was 7-KC. After the storage period, oxysterols in powdered milk reached 1.81% of total cholesterol.  The most stable cholesterol was in the milk–egg mixture and its oxidation was the slowest. This study showed the presence of COPs in milk powder, egg powder and milk–egg powder and the effect of storage on cholesterol oxidation.

## Introduction

Cholesterol (the major sterol in mammals) is a monounsaturated sterol, a lipid molecule in cell membranes and lipoproteins. Fats account for 3.7% of the 12.6% of milk solids. Cholesterol is the major sterol in milk (95% of sterols) and is mainly found in the membrane of milk fat globules^1,2^. Cholesterol plays an important regulatory role as a precursor of steroid hormones, bile acids and vitamin D. Due to its structure (27 carbons and a single double bond at 5, 6 position), cholesterol is highly susceptible to oxidation. Oxidation of cholesterol leads to the formation of bioactive molecules named oxysterols^3–5^.

**Figure 1:**
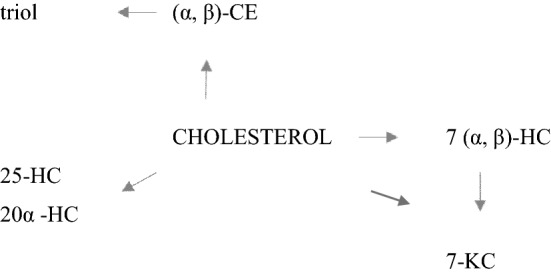
Simplified scheme of oxysterols formation.

The oxidation process can be initiated by the oxidation of polyunsaturated fatty acids and catalyzed by light and temperature. Deprotonation of the carbon atom at position 7 by the attack of a reactive oxygen species promotes the formation of cholesterol oxidation products, including 7α and 7β-hydroperoxides (the most representative ones in the oxysterols’ family). Their degradation products include 7α-hydroxycholesterol (7α-HC), 7β-hydroxycholesterol (7β-HC) and 7-ketocholesterol (7-KC), which can be formed from 7 (α, β)-HC. When a free radical attacks the double bond between carbon atoms at positions 5 and 6, α- and β-epoxycholesterol (α-CE and β-CE) are formed which are hydrated by the formation of cholestantriol (triol)^6^. Furthermore, side-chain oxidation generates 20α- and 25-hydroxycholesterol (20α-HC and 25-HC) (Fig. 1).

Autoxidation of cholesterol leads to the formation of hydroperoxides and the next various cholesterol oxidation products (COPs). Oxysterols are similar in structure to cholesterol but have an additional oxygen group. The oxidation of cholesterol is produced by non-enzymatic or enzymatic oxidation^7^. The most commonly detected oxysterols in foods are 7α-HC, 7β-HC, a product of their dehydrogenation 7-KC and α-CE, β-CE. The most biologically active and cytotoxic COPs include 25-HC, triol, 7α-HC, 7β-HC, 7-KC and epoxide-cholesterol derivatives^5,8^. It is known that 7-KC (> 30% of total COPs) is used as an index of cholesterol oxidation. It is readily formed and can be absorbed through the ingestion of cholesterol-rich foods, as cholesterol can be obtained in the body from diet or can be synthesized de novo^9^.

Numerous studies have shown that oxidation products (COPs) in foods can reach 1% of total cholesterol and occasionally 10% or more^1^. The increased temperature during food preparation and heating promotes the oxidation process of cholesterol^3,10^. According to Zmysłowski and Szterk^11^ oxysterols have the property of causing the formation of atherosclerosis and accelerating its progression.

As mentioned above, oxysterols can be derived to human organism mainly through diet (heat-treated or processed food), in vivo conversion of sterols to oxysterols in cells, blood and tissues, and in vivo oxidation of skin cosmetics with phytosterols. Oxysterols from food can be absorbed at different rates depending on the length of their side-chain. In the human organism, COPs can follow different pathways: Secretion into the intestinal lumen, esterification and distribution by lipoproteins into different tissues or degradation mainly in the liver^12^. It needs to be emphasized that a certain type of food can lead to the accumulation of COPs and cytotoxic, apoptotic and proinflammatory effects^13^. The main dietary sources of oxysterols are eggs and egg-derived products, thermally processed milk and milk-derived products, fried meat, and also vegetables fried by frying oil^7^.

Current evidence indicates that oxysterols are mutagenic, carcinogenic, cytotoxic, and immunosuppressive. They also inhibit DNA/biosynthesis of cholesterol and impair cell membrane functionality. Recent data suggest that oxysterols play an important role in neurodegenerative diseases like Huntington's disease, Alzheimer's and Parkinson's diseases, as well as in the development of cancer^6,10,14–17^.

An increased interest in the sources of oxysterols in the human organism is observed. In the case of the biological role of cholesterol oxidation products, the qualitative evaluation, monitoring and prevention of the oxysterols’ formation during the processing and storage of animal-based foods should be a key issue.

Studies of oxysterols in milk and powdered eggs are often undertaken, however, the range of oxysterols tested is usually limited. It should be pointed that milk and egg powders are used by product developers to formulate new powdered confectionery products. Powders are often rehydrated and added as an ingredient to the other products. These products are heat-treated (e.g. baking, frying), which can result in high amounts of oxysterol. Therefore, the present study aimed to compare cholesterol and cholesterol oxidation products in milk, egg and milk–egg powders after production and during storage.

## Results

### Composition and pH

The analysis of the physicochemical properties of raw milk, raw egg and milk–egg blend are presented in Table 1. The results show that raw milk has the highest moisture content compared to raw egg and milk–egg blend. Chemical composition of fat, total protein, ash and acidity is a specific for product and varies considerably (P < 0.05).

**Table 1:** Composition (g 100 g^−1^) and acidity characteristics of raw milk, raw egg and milk–egg blend.

Parameters	Milk	Egg	Milk–egg blend
Moisture	88.30 ± 0.15^a^	75.84 ± 0.17^b^	83.00 ± 0.10^c^
Fat	3.21 ± 0.22^a^	10.78 ± 0.66^c^	6.66 ± 0.44^b^
Total protein	3.32 ± 0.30^a^	12.68 ± 1.03^c^	7.24 ± 0.09^b^
Ash	0.78 ± 0.03^a^	0.90 ± 0.08^a^	0.83 ± 0.14^a^
pH	6.71 ± 0.05^b^	6.60 ± 0.02^a^	6.66 ± 0.05^ab^

^a,b^—different letters in the row denote statistically significant differences between the results (P < 0.05); ± means SD.

Good quality raw materials and optimized parameters used for spray-drying make the production of powders with low moisture content possible. Many oxidation reactions that occur in foods use water as a media or substrate. The presence of water molecules significantly influences lipid oxidation and increases the formation of COPs by facilitating the movement of the molecules^18^. According to Kulig et al.^19^ the oxidation of cholesterol is accelerated by the presence of water and salts.

The drying (thermal) treatment resulted in significant chemical modifications in the processed milk and egg as shown in Table 2. Milk powder moisture of 2.50 (g 100 g^−1^) is consistent with values obtained by Chudy and Makowska^20^ and met Codex Alimentarius^21^ requirements for whole milk powder. At the same time, the obtained value for the moisture powder milk was lower than egg powder and milk–egg powder.

**Table 2:** Composition (g^.^100 g^−1^) and acidity characteristics of raw milk powder, egg powder and milk–egg powder.

Parameters	Milk powder	Egg powder	Milk–egg powder
Moisture	2.50 ± 0.04^a^	3.10 ± 0.07^b^	3.00 ± 0.10^b^
Fat	26.50 ± 0.82^a^	43.03 ± 1.66^c^	37.18 ± 1.44^b^
Total protein	27.55 ± 0.93^a^	50.68 ± 1.03^c^	38.94 ± 2.09^b^
Ash	6.11 ± 0.22^c^	3.80 ± 0.02^a^	4.63 ± 0.18^b^
pH	6.56 ± 0.04^a^	7.20 ± 0.08^c^	6.66 ± 0.05^b^

^a,b^—different letters in the row denote statistically significant differences between the results (P < 0.05); ± means SD.

The results show that the fat content of powder milk, egg powder and milk–egg powder ranged from 26 to 42 (g 100 g^−1^) and the water content was less than 5%. According to Abreha et al.^22^, the low moisture (< 5%) and water activity (a_w_ ~ 0.4–0.5) of egg powders make unfavourable conditions for microbial growth. The fat content of powder milk (26.50 g 100 g^−1^) corresponds to the values obtained by Pugliese et al.^23^ and the protein content of 27.55 (g100 g^−1^) corresponds to the values obtained by Magan et al.^24^. The analysis of physicochemical properties showed statistically significant differences (P < 0.05) in acidity within powder milk, egg powder and milk–egg powder. The differences in pH between raw materials and regenerated powders may result from changes in the properties of proteins (including their buffering properties) under the influence of temperature during the drying process. Acidity influences the type of oxysterols formed at pH between 5.5 and 3.0, the β-isomer and is more acid labile than the α-isomer, preferentially converting into a triol by hydrolysis^25^. Abreha et al.^22^ showed in their study that egg powders have a low water activity of 0.4 and acidity between 7.5 and 8.5 pH.

### Cholesterol and cholesterol oxidation products

After production, the mean cholesterol content in milk powder was 0.2 g 100 g^−1^ fat (result of calculation) and in egg powder was 3.4 g 100 g^−1^ (Table 3). Similar contents were previously obtained by Chitra et al.^26^ – 0.4 g 100 g^−1^ of fat and – 2.3 g 100 g^−1^ fat of analysed powdered milk and powdered eggs, respectively. There were no statistically significant changes in the cholesterol content of each type of powder during storage (P ˃ 0.05). This effect was also observed in the studies of Singh and Gallier^27^ on the cholesterol oxidation in egg powder. The authors showed that five cholesterol oxides increased during 12-months storage, although the cholesterol content remained constant.

**Table 3:** Cholesterol content (mg g^−1^ powder) of powders.

Storage time [months]	Milk powder	Egg powder	Milk–egg powder
0	0.56 ± 0.04^a^	14.56 ± 0.44^c^	5.55 ± 0.27^b^
3	0.54 ± 0.03^a^	14.56 ± 0.73^c^	5.58 ± 0.22^b^
6	0.55 ± 0.04^a^	14.49 ± 0.59^c^	5.54 ± 0.30^b^
12	0.53 ± 0.02^a^	14.46 ± 0.58^c^	5.53 ± 0.29^b^
24	0.49 ± 0.03^a^	14.33 ± 1.00^c^	5.39 ± 0.22^b^

^a,b^—different letters within the column denote statistically significant differences between the results (P < 0.05); ± means SD.

It was obvious that the largest amounts of oxysterols would be formed quantitatively in egg powder, owing to its highest cholesterol content. However, the calculated share in COPs to cholesterol (assuming that the cholesterol content was 100%) demonstrated that the cholesterol in milk powder was the most susceptible to the oxidation processes. The percentage changed from 0.11 to 1.81%, which may be due to the fact that cholesterol in milk is mainly located in the milk fat globule membrane (MFGM) and it is prone to substantial damage in dairy processing. According to Mazalli and Bragagnolo^28^ this change increases its susceptibility to oxidation. The rate of cholesterol oxidation in egg powder was slower than in milk powder and in milk–egg powder. The initial COPs content of cholesterol was 0.03%, while after the storage it was 0.72%. In the work of Caboni et al.^29^, the oxidation affected about 0.6% of the cholesterol in egg powder after 12 months of storage at 20 °C. The cholesterol in milk–egg powder was quite stable. Initially, the content of COPs with cholesterol was 0.03%, which finally reached 0.58%.

The oxysterols analysed in this study were grouped according to their formation pathways (Tables 4–6). Cholesterol oxidation occurs primarily through free radicals under the influence of heat. The easiest to accomplish is the oxidation of carbon at the C-7 position^1^. Heating during technology processes (pasteurisation, spray drying) first causes the formation of all 7(α, β)-HC and 7-KC but also 7β-CE, indicating a double oxidation mechanism by atmospheric oxygen and free radicals. In milk powder, egg powder and milk–egg powder, the content of the first group of oxysterols (the sum: 7α-HC, 7β HC, 7-KC) immediately after production was 0.300 µg g^−1^ powder, 1.837 µg g^−1^ powder and 1.493 µg g^−1^ powder, respectively. After 24 months, it increased to 4.513 µg g^−1^ milk powder, 70.246 µg g^−1^ egg powder and 109.726 µg g^−1^ milk–egg powder. After 6 months of storage, the dominant oxysterol in milk and egg powders was 7α-HC and in milk–egg powder — 7-KC. They accounted for 28.7%, 33.6% and 54% of the sum of all oxysterols in a given powder, respectively (Table 4).

**Table 4:** Content (µg g^−1^ powder) of 7α-HC, 7β-HC, 7-KC in powders.

Storage time [months]	Milk powder	Egg powder	Milk–egg powder
**7α-HC**			
0	0.011 ± 0.001^a^	0.949 ± 0.056^c^	0.425 ± 0.041^a^
3	0.625 ± 0.043^e^	1.983 ± 0.206^d^	1.675 ± 0.121^e^
6	0.968 ± 0.079^f^	8.633 ± 0.777^h^	1.247 ± 0.120^d^
12	0.720 ± 0.088^e^	6.786 ± 0. 599^g^	3.343 ± 0.290^h^
24	1.360 ± 0.099^g^	30.521 ± 2.001^j^	9.766 ± 0.554^j^
**7β-HC**			
0	0.077 ± 0.010^b^	0.815 ± 0.009^b^	0.675 ± 0.056^c^
3	0.178 ± 0.090^c^	2.093 ± 0.158^de^	1.620 ± 0.166^e^
6	0.407 ± 0.041^d^	6.869 ± 0.545^g^	1.128 ± 0.175^d^
12	0.728 ± 0.073^e^	11.932 ± 1.001^i^	2.339 ± 0.117^g^
24	1.558 ± 0.111^g^	35.525 ± 3.332^j^	9.447 ± 0.771^j^
**7-KC**			
0	0.212 ± 0.003^c^	0.073 ± 0.003^a^	0.400 ± 0.023^a^
3	0.400 ± 0.031^d^	0.977 ± 0.101^c^	1.829 ± 0.201^f^
6	0.204 ± 0.040^c^	2.448 ± 0.166^e^	6.446 ± 0.476^i^
12	0.339 ± 0.021^d^	5.179 ± 0.047^g^	0.401 ± 0.040^a^
24	1.594 ± 0.143^g^	4.197 ± 0.078^f^	0.513 ± 0.043^b^

^a,b^—different letters within the column denote statistically significant differences between the results (P < 0.05); ± means SD.

The second group of oxysterols was α-CE, β-CE and triol. There was observed the higher increase of α-CE than other COPs after 12 months of storage in all powders. In addition, the highest statistically significant increase in β-CE was observed after 24 months of storage (Table 5). According to Caboni et al.^29^ the sum of 7α-HC and 7β-HC represented 52% of total COPs at the end of the storage at room temperature. At the same stage, the sum of α-CE, β-CE and triol were about 35% of the total COPs.

**Table 5:** Content (µg g^−1^ powder) of α-CE, β-CE and triol in powders.

Storage time [months]	Milk powder	Egg powder	Milk–egg powder
**α-CE**			
0	0.298 ± 0.038^cd^	0.759 ± 0.045^e^	0.311 ± 0.029^f^
3	0.324 ± 0.015^d^	0.946 ± 0.064^f^	0.100 ± 0.009^c^
6	0.672 ± 0.025^f^	1.089 ± 0.097^g^	0.836 ± 0.048^g^
12	1.346 ± 0.135^g^	5.635 ± 0.444^j^	1.689 ± 0.143^i^
24	0.297 ± 0.017^cd^	3.727 ± 0.382^i^	2.307 ± 0.199^j^
**β-CE**			
0	0.000 ± 0.000^a^	1.294 ± 0.100^gh^	0.050 ± 0.010^b^
3	0.260 ± 0.011^c^	1.363 ± 0.047^h^	1.905 ± 0.179^i^
6	0.189 ± 0.020^b^	5.362 ± 0.323^j^	1.252 ± 0.063^h^
12	0.442 ± 0.039^e^	7.182 ± 0.570^k^	1.193 ± 0.082^h^
24	1.735 ± 0.087^h^	25.822 ± 2.066^l^	6.039 ± 0.546^k^
**Triol**			
0	0.000 ± 0.000^a^	0.310 ± 0.009^a^	0.024 ± 0.003^a^
3	0.286 ± 0.018^c^	0.303 ± 0.024^a^	0.175 ± 0.008^d^
6	0.745 ± 0.067^e^	0.413 ± 0.058^b^	0.262 ± 0.019^e^
12	0.315 ± 0.022^d^	0.662 ± 0.043^d^	1.859 ± 0.201^i^
24	0.649 ± 0.054^f^	0.517 ± 0.014^c^	1.300 ± 0.092^h^

^a,b^—different letters within the column denote statistically significant differences between the results (P < 0.05); ± means SD.

The cholesterol oxidation of the side chain was much slower. Oxysterols of the third group (20α-HC and 25-HC) were not found in any of the three powders immediately after production. After 24 months, the sum of oxysterols of the third group in milk powder, egg powder and milk–egg powder was 1.776 µg g^−1^ powder, 3.714 µg g^−1^ powder and 2.132 µg g^−1^ powder, respectively (Table 6). Caboni et al.^29^ after 12 months of storage at 20 °C found detectable amounts of 25-HC (2.0 μg g^−1^ fat) in egg powder samples.

**Table 6:** Content (µg g^−1^ powder) of 20α-HC and 25-HC in powders.

Storage time [months]	Milk powder	Egg powder	Milk–egg powder
**20α-HC**			
0	0.000 ± 0.000^a^	0.000 ± 0.000^a^	0.000 ± 0.000^a^
3	0.000 ± 0.000^a^	0.036 ± 0.008^b^	0.000 ± 0.000^a^
6	0.197 ± 0.017^b^	0.596 ± 0.048^d^	0.338 ± 0.031^b^
12	0.992 ± 0.086^c^	1.155 ± 0.119^e^	0.543 ± 0.045^c^
24	1.537 ± 0.137^d^	2.330 ± 0.112^f^	1.833 ± 0.146^d^
**25-HC**			
0	0.000 ± 0.000^a^	0.000 ± 0.000^a^	0.000 ± 0.000^a^
3	0.000 ± 0.000^a^	0.000 ± 0.000^a^	0.000 ± 0.000^a^
6	0.000 ± 0.000^a^	0.252 ± 0.038^c^	0.324 ± 0.043^b^
12	0.000 ± 0.000^a^	0.675 ± 0.065^d^	0.525 ± 0.042^c^
24	0.239 ± 0.031^b^	1.384 ± 0.140^e^	0.299 ± 0.025^b^

^a,b^—different letters within the column denote statistically significant differences between the results (P < 0.05); ± means SD.

## Discussion

In recent years, scientific studies provide clear evidence that oxysterols are formed in foods during their processing and storage.  Food oxysterols are mainly non-enzymatic origin but the enzymatic pathway for oxysterols conversion or formation is also possible. ^10^. Oxysterols ingested in food are absorbed and packed into lipoproteins that are taken up by hepatic cells. It should be noted that oxysterols affect the nutritional profile of foods and are also associated with the development of various diseases (Alzheimer’s disease, atherosclerosis, bowel disease), as well as cancer and ageing^30,31^.

Interest in oxysterols has attracted two major audiences in food science: Food technologists and nutritionists. From a nutritional point of view, 7-HC deserves attention due to its high concentration in the human body. There are two isomeric forms of 7-HC: α and β. The α isomer is produced in the enzymatic pathway and it is involved in the synthesis of bile acids. The isomer β is formed by reactions with oxygen radicals. Non-enzymatically,7β-HC has strong cytotoxic properties and is implicated in various pathological states^32^. This study shows that after 12 months of storage, the content of 7β-HC in powdered milk, powdered egg and powdered milk–egg increased and were respectively 1.373 µg g^−1^ cholesterol for powder milk, 0.825 µg g^−1^ cholesterol for powdered egg and 0.422 µg g^−1^ cholesterol for powdered milk–egg. It is supposed that polyunsaturated fatty acids may be responsible for inhibiting the oxidation of cholesterol and the formation of small amount of oxysterols in relation to the cholesterol content of egg powder. Their protective effect against cholesterol by attracting oxidation mechanisms was pointed out, among others, Innosa et al.^33^, who studied cholesterol oxidation and 7-KC formation in boiled eggs and cakes.

Also Risso et al.^34^ analysed the 7β-HC changes in milk powder during storage. They found that the content of 7β-HC reached 0.265 µg g^−1^ cholesterol and 0.569.83 µg g^−1^ in skim milk powder and whole milk powder, respectively, after 12 months, and these amounts are potentially toxic in several in vitro and in vivo experimental studies.

Angulo et al.^35^ examined the presence of eight oxysterols in fresh and 6 months stored powdered milk. The samples were stored in closed polystyrene bags at room temperature. The sum of C7 cholesterol oxides (7α-HC, 7β-HC, 7-KC) in milk powders after production was 0.4 µg g^−1^ powder, which is consistent with the results of this study (0.3 µg g^−1^ powder). As recently reviewed by Chang et al.^36^ the addition of small quantities of dietary 7-KC contributed to accelerated hepatic steatosis and inflammation in obese mice models.

In conclusion, a mixture of raw milk and eggs in the ratio of 1.4:1 proved to be a protective matrix for cholesterol. The oxidation of cholesterol was slowest in milk–egg powder, followed by egg powder, and most dynamic in milk powder. Measurement of oxysterols after the production of powders is a useful tool for monitoring the nutritional value of powders. It is significant to control toxic compounds in food to avoid their adverse effects on human health. The results obtained by researchers on oxysterol content in powders can be only partially compared. This is because the production conditions of the powder are usually not comparable, as different temperatures are used in the production processes and storage, as well as packaging materials. Frequently, the materials studied are market-available powders for which there is no information on the technological production conditions. Additionally, in many publications, less than eight oxysterols are analysed. Hence, each research is different and provides valuable new data and insights.

## Methods

### Powder production

**Figure 2:**
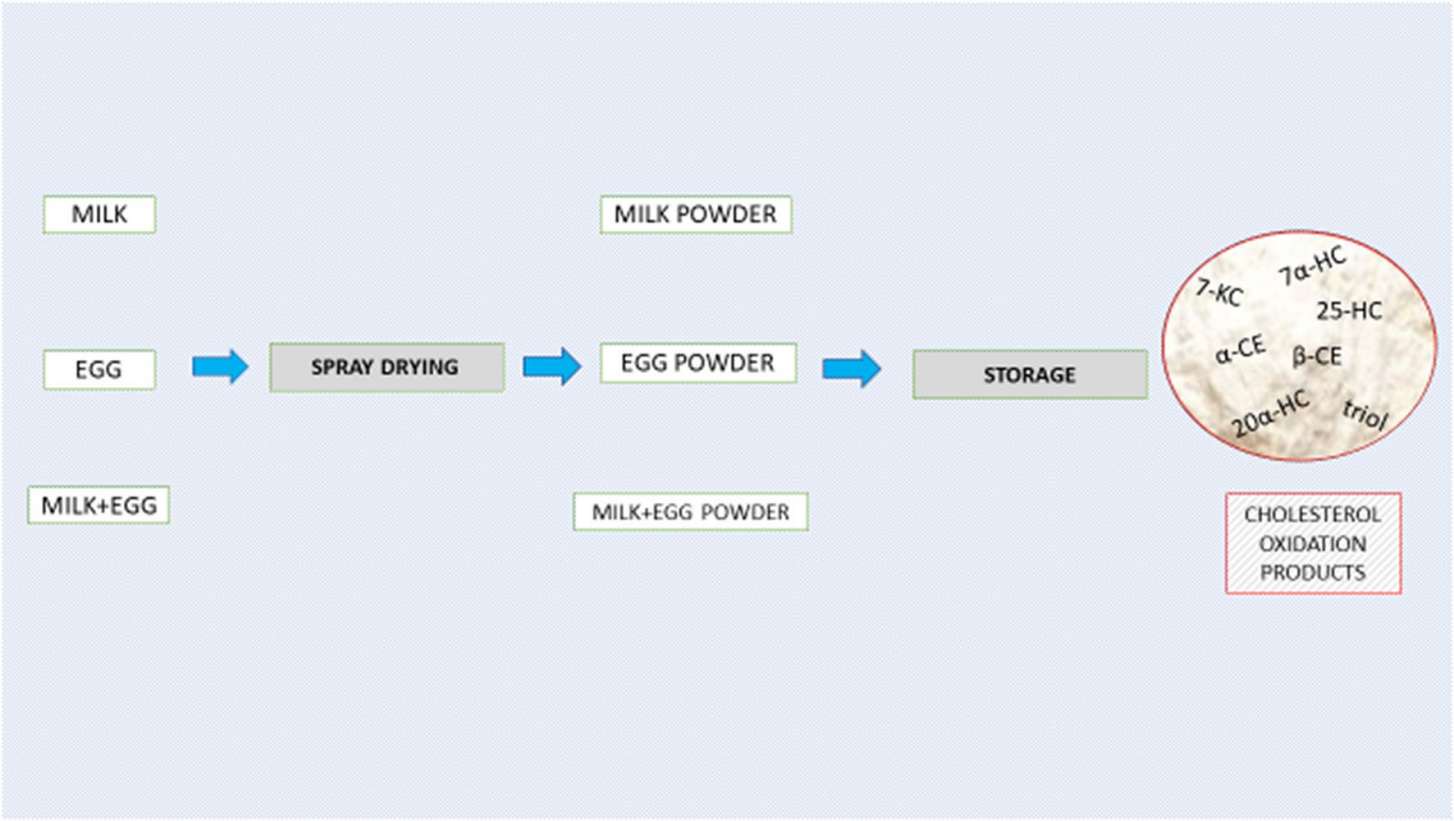
The scheme of experiment.

The model powder was designed as a component for the production of powdered cakes, and was prepared by combining liquid milk and liquid eggs (1.4:1 w/w). Raw milk and fresh eggs were used in the experiment. The eggs were cleaned by washing, and allowed to dry, carefully deshelled and mixed with milk. The mixture was heated to 55 °C in a container (Spomasz, Poland), homogenized for 10 min at 18 MPa in Rannie homogenizer (Denmark) and pasteurized for 30 min at 60 °C in Alfa Laval (Sweden) plate heat exchanger. The liquid mass was dried (inlet temperature of the drying air was 170 °C and outlet temperature was 75 °C) in GEA Niro spray dryer (Denmark). After cooling, 100 g of the samples were packaged (Mulitvac A 300/16) in plastic bags (sized 10 cm × 15 cm). Packing film was polyethylene terephthalate–polyethylene laminate (Akerlund & Rausing, Poland), with water vapor permeability of 12 g m^−2^ per 24 h and oxygen permeability of 73 cm^3 ^m^−2^ per 24 h at 0.1 MPa. The powder was produced in the experimental plant of the University of Life Sciences in Poznań (Poland). Powders were stored in the absence of light at temperature of 20 ± 1 °C (relative humidity of max. 75%) for a period of 24 months. Full fat milk powder and egg powder were produced following the same procedures. The scheme of the experiment is given in Fig. 2.

### Composition and pH

All solvents and reagents were of analytical grade and purchase from Merck (Darmstad, Germany).

The total solid moisture was determined according to GEA Niro Method with modifications^37^. The weighing dish was dried and cool it in desiccator. The test sample (approx. 3 g) was weighed, mixed with dried sand and kept in a drying oven set at 102 °C for 3 h. Following this, the sample was cooled in the desiccator before the second weighing. This procedure was repeated but drying only 1 h instead of 3 h until the difference between two subsequent weights was 0.0002 g or less. Moisture = [(b − c)/(b − a)] × 100%, where: a—weight of empty dish, b—weight of dish + powder, c—weight of dish + dried powder.

In order to determine the fat content, the ISO 1736^38^ method was applied. The sample was treated with ammonia and ethyl alcohol. Fat was extracted with diethyl ether and petroleum ether. Mixed ethers were evaporated and the residue was weighed. This is usually known as the Rose-Gottlieb principle.

The protein content was determined by measuring total nitrogen, using the Kjeldahl method^39^. To convert the Kjeldahl nitrogen reading to cheese protein, the nitrogen reading was multiplied by a factor of 6.38.

Determination of ash was conducted^40^. About 2 g of powder was weigh into the dish spreading the powder uniformly in the dish before weighing. Sample was heat in the muffle furnace until the powder was completely charred and smoke was no longer given off. The ash was cool in a desiccator for 60 min and weigh. The result was presented in percentage.

The pH was measured in the liquid semi-products and reconstituted powders using a CP-411 pH meter (Elmetron, Zabrze, Poland).

### Cholesterol and cholesterol oxidation products

In order to determine the cholesterol content, the sample of powder (1.000 g) was dissolved in 10 ml of water, 0.1 ml 5α-cholestane (internal standard) and 50 ml of chloroform/methanol mixture (2:1, v/v) were added. The sample was shaken (15 min), filtered and transferred into a separatory funnel with 15 ml of water. The lower, chloroform layer was filtered over anhydrous sodium sulfate. 1 ml of anhydrous ethanol was added and the sample was evaporated to dryness under nitrogen. Lipids were saponified with 1 M KOH in methanol (18 h at room temperature), then unsaponifiables were extracted with hexane/methyl tert-butyl ether (Sigma-Aldrich, St. Louis, MO, USA) (1:1, v/v). The solvent was evaporated under nitrogen atmosphere. After silylation by Sylon BTZ (Supelco, Bellefonte, PA, USA) cholesterol was separated using gas chromatograph HP 6890 equipped with a DB-5MS capillary column (30 m × 0.25 mm × 0.25 mm; J&W Scientific, USA). Helium was used as a carrier gas at a flow rate of 1 ml min^−1^. Analyses were performed isothermally at 290 °C, and injector and detector temperatures were set at 320 °C. Samples were injected using a split ratio of 1:10. Cholesterol identification was based on the retention data^41^.

Cholesterol oxidation products (7α-HC, 7β-HC, 7-KC, α-CE, β-CE, triol, 20α-HC and 25-HC) were determined according the methodology by Przygoński et al.^42^. The sample of powder (1.000 g) was dissolved in 10 ml of water, 0.1 ml 19-hydroxycholesterol (internal standard) and 50 ml of chloroform/methanol mixture (2:1) containing BHT (0.050 ml/ml) were added. The sample was shaken (15 min), filtered and transferred into a separatory funnel with 15 ml of water. The chloroform layer was filtered over anhydrous sodium sulfate. 1 ml of anhydrous ethanol was added and the sample was evaporated to dryness under nitrogen. Lipids were saponificated with 2 ml of sodium methylate (10%) mixed with MTBE (4:6, v/v), left for 1 h and transferred to the centrifuge tube together with 4 ml of water and 5 ml of chloroform. The mixture was centrifuged (2000 rpm), after which water layer (upper) was discarded. Then, 2 ml 1% citric acid was added to the tube and shaken and centrifuged again. The water layer was discarded and the chloroform extract, after addition of 1 ml of anhydrous ethanol, was evaporated under nitrogen to dryness. Sample was dissolved in 0.250 ml of chloroform and transferred onto the SPE-PAK column. Oxysterols was eluted with 7 ml of acetone. Solvent was evaporated in a stream of nitrogen and mix of anhydrous pyridine (0.1 ml) and BSTFA + 1% TMCS (0.1 ml) was added. Vials were flushed with nitrogen, capped and delicately shaken. After 4 h samples were ready for the analysis. COPS were separated using gas chromatograph HP 6890 using FID detector and DB-5MS capillary column (30 m × 0.25 mm × 0.25 mm; J&W Scientific, USA). Analysis parameters were following: 1 min at 60 °C, then temperature was risen to 270 °C at 25 °C min^−1^ and to 290 °C at 2.5 °C min^−1^. Helium was used as a carrier gas at a flow rate of 1 ml min^−1^. Injector and detector were held at 300 °C, split ratio was set at 1:40. Oxysterols were identified based on the retention times. The compounds were previously verified by mass spectrometry utilizing our library and published data by Gorassini et al.^43^.

The recovery of COPs was 94.3% (7β-HC) to 99.9% (7α-HC). The amount of COP was calculated using internal standard 19-hydroxycholesterol. The analytical standards used for identification of COPs were supplied by Sigma-Aldrich (Munich, Germany) and 19-hydroxycholesterol from Steraloids Inc. (Newport, RI, USA), 5α-cholestane from Sigma-Aldrich (Deisenhofen, Germany). Purity of standards 5α-cholestane and 19-hydroxycholesterol was 95%. Silylation mixture of BSTFA (N,O-bis(trimethylsilyl)trifluoro acetamide) with 1% TMCS (trimethylchlorosilane) was obtained from Fluka Chemie (Buchs, Switzerland). Sep-Pak amino cartridges were obtained from Waters (Milford, Massachusetts, USA). Analyses of cholesterol and cholesterol oxidation products (COPs) were performed directly after the production process and after 3, 6, 12 and 24 months of storage.

### Statistical analyses

The choice statistical significance was based on analysis of univariate (ANOVA); the Fisher test was used. The differences were considered significant at P < 0.05. The statistical calculations were carried out using Statistica data analysis software, version 10 (StatSoft, Oklahoma, US). In the tables, means (n = 3), standards deviation and statistically significant differences are presented.
